# Changes in Incidence and Survival by Decade of Patients With Primary Colorectal Lymphoma: A SEER Analysis

**DOI:** 10.3389/fpubh.2020.486401

**Published:** 2020-10-16

**Authors:** Qingguo Li, Shaobo Mo, Weixing Dai, Yaqi Li, Ye Xu, Xinxiang Li, Guoxiang Cai, Sanjun Cai

**Affiliations:** ^1^Department of Colorectal Surgery, Fudan University Shanghai Cancer Center, Shanghai, China; ^2^Department of Oncology, Shanghai Medical College, Fudan University, Shanghai, China

**Keywords:** primary colorectal lymphoma, incidence, survival analysis, treatment, SEER

## Abstract

**Purpose:** To reveal changes in the incidence, treatment, and survival of patients with colorectal lymphoma.

**Methods:** Patients diagnosed with primary colorectal lymphoma (PCL) or lymphoma between 1973 and 2014 were identified in the SEER registry. The incidence was estimated by age and join-point analysis. The incidence of different subtypes and the surgical resection rates were compared over different time periods.

**Results:** The PCL incidence increased from 1.4 per 1 000 000 people in 1973 to 3.5 in 2014, with an annual percentage change (APC) of 1.98% (95% confidence interval [CI]: 1.29–2.68%, *P* < 0.001) from 1985 to 2014. No statistically significant change was found between 1973 and 1984. For people younger than 60 years, there was a slight increase in PCL incidence, from 0.6 to 1.4%, from 1973 to 2014. For people age 60 or older, there was a statistically significant increase in PCL incidence from 5.4 to 14.1% over the same time period. The 5-year cause-specific survival (CSS) for PCL improved markedly from 41.6% in the period 1973–1976 to 80.2% in the period 2009–2012 (*P* < 0.001). Conversely, the proportion of patients who received surgical therapy decreased gradually from 83.3–100 to 47.7–52.6% throughout the studied time period.

**Conclusions:** The incidence of PLC has increased in recent decades. The 5-year CSS of PCL increased continuously, while the rate of surgical resection decreased steadily. These changes in survival trends and therapy strategies indicate that PCL can be well-managed with newer therapeutic reagents.

## Introduction

Lymphoma is the seventh-most common cancer type in the United States and is the seventh-leading cause of cancer-related deaths among both men and women. The incidence of lymphoma has been increasing in recent decades (1, 2). Approximately 40% of lymphomas have extranodal manifestations, and the most common site of extranodal involvement is the gastrointestinal tract (3). The incidence of primary colorectal lymphoma (PCL) is rare, accounting for only 0.2–1.2% of all colorectal malignancies (4). The most common variety of colonic lymphoma is non-Hodgkin's lymphoma (NHL) (5). Therapeutic strategies for NHL include radical tumor resection plus multiagent chemotherapy for early-stage patients, and biopsy plus multidrug chemotherapy for advanced stage patients.

Due to the low incidence of PCL, most prior studies of this disease were conducted with small sample populations and at a single institution (6–8). Contemporary population-based studies of PCL have revealed clinical and demographic patient factors that may be associated with the prognosis of colorectal lymphomas (9, 10). Chouhan et al. (9) focused on all gastrointestinal tract lymphomas, while Cai et al. (10) limited their study to factors that were associated with prognosis after the surgical resection of PCL. In the current study, we investigated the epidemiological changes of colorectal lymphoma from 1973 to 2014 by analyzing data from the Surveillance, Epidemiology, and End Results (SEER) database. We also reported the incidence by lymphoma subtype and trends in the use of initial surgical therapy for these patients.

## Methods

### Data Collection

The SEER Cancer Statistics Review (http://seer.cancer.gov/data/citation.html) is a report on the most recent cancer incidence, mortality, survival, prevalence, and lifetime risk statistics and is published annually by the Data Analysis and Interpretation Branch of the National Cancer Institute, USA. Two cohorts in the SEER database were used in the present study. The cohort of the SEER 9 registry from 1973 to 2014 was used to estimate the long-term incidence of systematic lymphoma and PCL, as previously described (11). A second cohort, the SEER 18 registry, which covers 28% of the US population, was created to estimate the incidence by histologic subtype, the use of surgical therapy, and survival. To ensure sufficient follow-up time, patients diagnosed after 2012 were excluded from the study.

We extracted all data from cases of lymphoma and lymphoma subtypes from the SEER database. Patients had to have a pathological diagnosis of a lymphoid neoplasm for inclusion. The site of the primary tumor was the colon (including cecum, appendix, right colon, hepatic flexure, transverse colon, splenic flexure, left colon, and sigmoid colon) and the rectum (overlapping lesions and rectum). For PCL, the site codes representing “colon” (C18.0–C18.9); “rectosigmoid junction” (C19.9), and “rectum” (C20.9) according to the *Third Edition of International Classification of Diseases for Oncology* (ICD-O-3) were used to identify eligible patients. The inclusion criteria in the current study were as follows: (1) Anatomic site of the primary tumor localized in the colon or rectum (ICD-O-3: C18.0–18.9, C19.9, and C20.9); (2) Histological type limited to lymphoma (ICD-O-3: 9590-9738); and (3) Malignant behavior (ICD-O-3 code: 3). The following subgroup types were included in this analysis because these types constitute the vast majority of cases: mantle cell lymphoma (ICD-O-3: 9673), diffuse large B-cell lymphoma (9680), Burkitt lymphoma (9687), follicular lymphoma (9690-9698), and marginal zone B-cell lymphoma (9699). The exclusion criteria were: (1) Patients without histological confirmation; and (2) Patients with unclear information regarding surgery. The lymphoma had to be the only primary or the first of multiple primaries.

We acquired permission to access the research data file in the SEER program through the National Cancer Institute, USA; the reference number was 10359-Nov2016. This study was approved by the ethics committee/institutional review board at Fudan University Shanghai Cancer Center.

### Statistical Analyses

Age-adjusted incidence was estimated as diagnoses per 1,000,000 patients per year (SEER^*^Stat software, version 8.3.21, Information Management Services, Inc., Calverton, MD). Changes in incidence were assessed and fit (Joinpoint Regression Program version 4.2, Information Management Services, Inc., Calverton, MD) using log-linear models and APCs estimated for the final, best-fitting model. Differences in trends were also assessed (11, 12).

Survival rates were generated using Kaplan-Meier curves, and the differences were compared with the log-rank test. Patients who were alive or dead due to other diseases at the last follow-up were excluded from analysis. The statistical evaluation was conducted with SPSS 22.0 (SPSS Inc., Chicago, IL, USA). All CIs are stated at the 95% confidence level. Statistical significance was defined as *P* < 0.05 (two-sided).

## Results

### PCL Incidence

**Figure 1:**
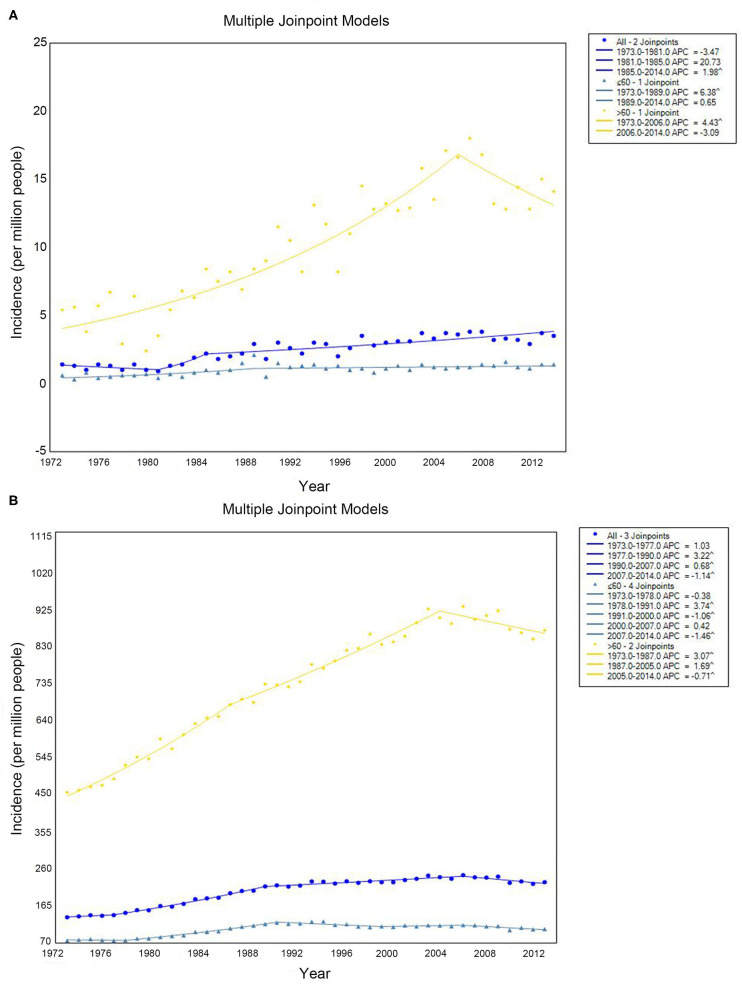
Incidence of primary colorectal lymphoma **(A)** and total lymphoma **(B)** by age. Incidence (per 1 000 000 persons) was calculated in SEER 9 registries, 1973–2014.

The PCL incidence increased from 1.4 per 1,000,000 people (hereafter, all incidence estimates are per 1,000,000 people) in 1973 to 3.5 in 2014, with an annual percentage change (APC) of 1.98% (95% confidence interval [CI]: 1.29–2.68%, *P* < 0.001) from 1985 to 2014 and no statistically significant change between 1973 and 1985 (Figure 1A, blue line). We then investigated whether there were differences in incidence between older and younger people according to a cutoff of age 60. For people younger than 60 years, there was a slight increase in PCL incidence from 0.6 to 1.4% from 1973 to 1989, with an APC of 6.38% (95% CI: 3.45–9.39%, *P* < 0.001), but no statistically significant change thereafter (Figure 1A, cyan line). For people 60 years or older, there was a statistically significant increase in PCL incidence from 5.4 to 14.1% from 1973 to 2014. The APC was 4.43% (95% CI: 3.50–5.36%, *P* < 0.001) from 1973 to 2006, with no statistically significant change thereafter (Figure 1A, yellow line).

The incidence of systemic lymphoma presentations increased from 136.1 to 226.2 with an APC of 3.22% (95% CI: 2.91–3.53%, *P* < 0.001) from 1977 to 1990, 0.68% (95% CI: 0.49–0.88%, *P* < 0.001) from 1990 to 2007, and −1.14% (95% CI: −1.81% to −0.46%, *P* = 0.002) from 2007 to 2014. There was no statistically significant change from 1973 to 1977 (Figure 1B, blue line). At same periods, for patients younger than 60, the APC for systemic lymphoma was 3.75% (95% CI: 3.30–4.19%, *P* < 0.001) from 1978 to 1991, −1.06% (95% CI: −1.84 to −0.27%, *P* = 0.01) from 1991 to 2000, and −1.46% (95% CI: −2.41 to −0.50%, *P* = 0.004) from 2007 to 2014. There was no significant change from 1973 to 1978 or from 2000 to 2007 (Figure 1B, cyan line). For patients 60 years or older, the incidence increased from 1973 to 1987 (APC 3.07%, 95% CI: 2.77–3.77%, *P* < 0.001) and from 1987 to 2005 (APC 1.69%, 95% CI: 1.47–1.92%, *P* < 0.001), but it decreased from 2005 to 2014 (APC −0.71%, 95% CI: −1.28 to −0.14%, *P* = 0.010) (Figure 1B, yellow line).

### PCL Incidence and Presentation by Subtype

**Figure 2:**
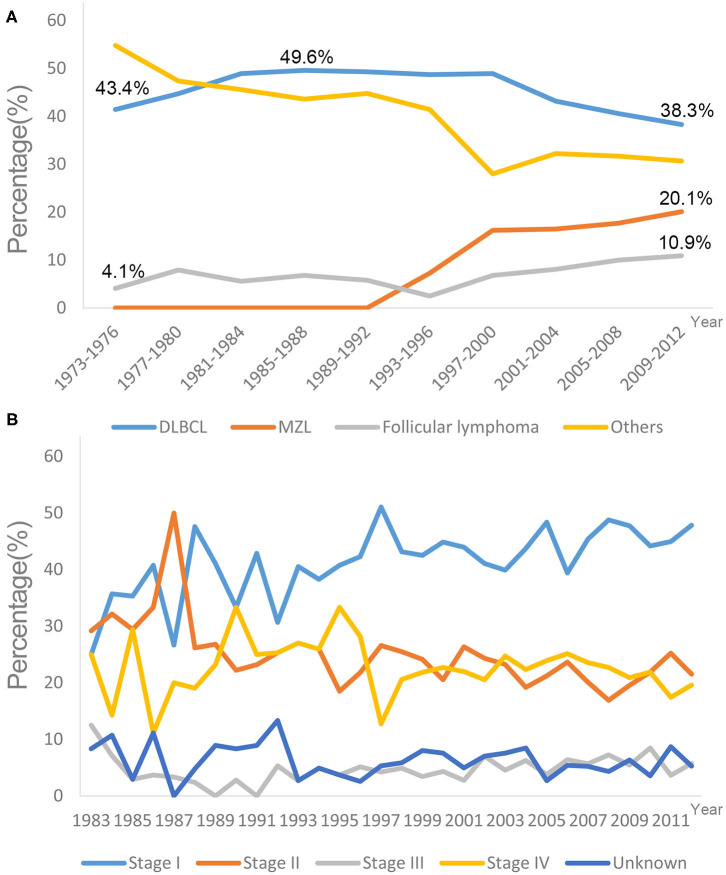
Trends in different lymphoma subtypes **(A)** and tumor stages **(B)** at diagnose in SEER 18 registries, 1973–2012. DLBCL, Diffuse large B-cell lymphoma; MZL, Marginal zone lymphoma.

A total of 3,860 eligible patients diagnosed with PCL between 1973 and 2012 were identified in the SEER 18 registry (Table 1). The majority of PCL cases were non-Hodgkin's lymphoma (99.2%), and the highest proportion was Diffuse large B-cell lymphoma-PCL (DLBCL-PCL) (43.4%), followed by extranodal marginal zone lymphoma (MZL) (14.2%) and follicular lymphoma (8.2%). Notably, the ratio of DLBCL in PCL increased gradually from 1973 to 1977, peaking at 49.6% in the period of 1985–1988 and decreasing to 38.3% in the period of 2009–2012. Conversely, the proportion of follicular lymphoma increased from 4.1% in 1973–1976 to 10.9% in 2009–2012. The unifying category of “extranodal MZL of mucosa-associated lymphoid tissue (MALT lymphoma)” was proposed more than two decades ago (13, 14) and was first recorded in 1995; it accounted for 20.1% of PCLs in the 2009–2012 period (Figure 2A).

**Table 1:** Patient and tumor characteristics of primary colorectal lymphoma diagnosed in SEER 18 registries, 1973–2012.

**Characteristic**	**No**.	**Percentage**
Sample size	3860	100
Median age, year	62	
Age at diagnosis		
Younger than age 60	1612	41.8
Age60 years older	2248	58.2
**Histology**		
Non-Hodgkin lymphoma	3831	99.2
B-cell lymphoma		
Diffuse large B-cell lymphoma	1675	43.4
Follicular lymphoma	317	8.2
Marginal zone lymphoma	550	14.2
Mantle-cell lymphoma	281	7.4
Burkitt lymphoma	241	6.2
Others	767	20.02
Hodgkin lymphoma	29	0.8
Stage (Ann Arbor Stage,1983+)		
I	1597	41.4
II	841	21.8
III	187	4.8
IV	826	21.4
Unknown^*^	409	10.6
Primary site		
Cecum	1322	34.2
Ascending colon	391	10.1
Hepatic flexure	73	1.9
Transverse	177	4.6
Splenic flexure	49	1.3
Descending	123	3.2
Sigmoid colon	385	10.0
Overlapping lesion	118	3.1
Rectosigmoid junction	104	2.7
Colon,NOS	390	10.1
Rectum	594	15.4

* *Unknown included 187 cases diagnosed before 1983 and was not staged according to Ann Arbor Stage*.

The most common primary site was the cecum (34.2%), followed by the rectum (15.4%). The median ages at diagnosis were 63, 65, and 63 years for DLBCL-PCL, MZL-PCL and follicular lymphoma, respectively. By comparison, the median age at diagnosis of MZL-PCL was significantly older than that for the other subtypes (*P* < 0.001, Mann-Whitney *U* test). Presentation with stage I disease occurred more frequently in MZL-PCL (67.09%) than in DLBCL-PCL (36.06%), follicular lymphoma (48.58%), and the other subtypes (35.66%) (*P* < 0.001, χ^2^ test). Specifically, there appeared to be no significant changes in the percentages of different tumor stages from 1983 to 2012 (Figure 2B). Because the Ann Arbor staging system was used only from 1983 onward, patients diagnosed before 1983 were not included in the stage analysis.

### PCL Initial Local Therapy

**Figure 3:**
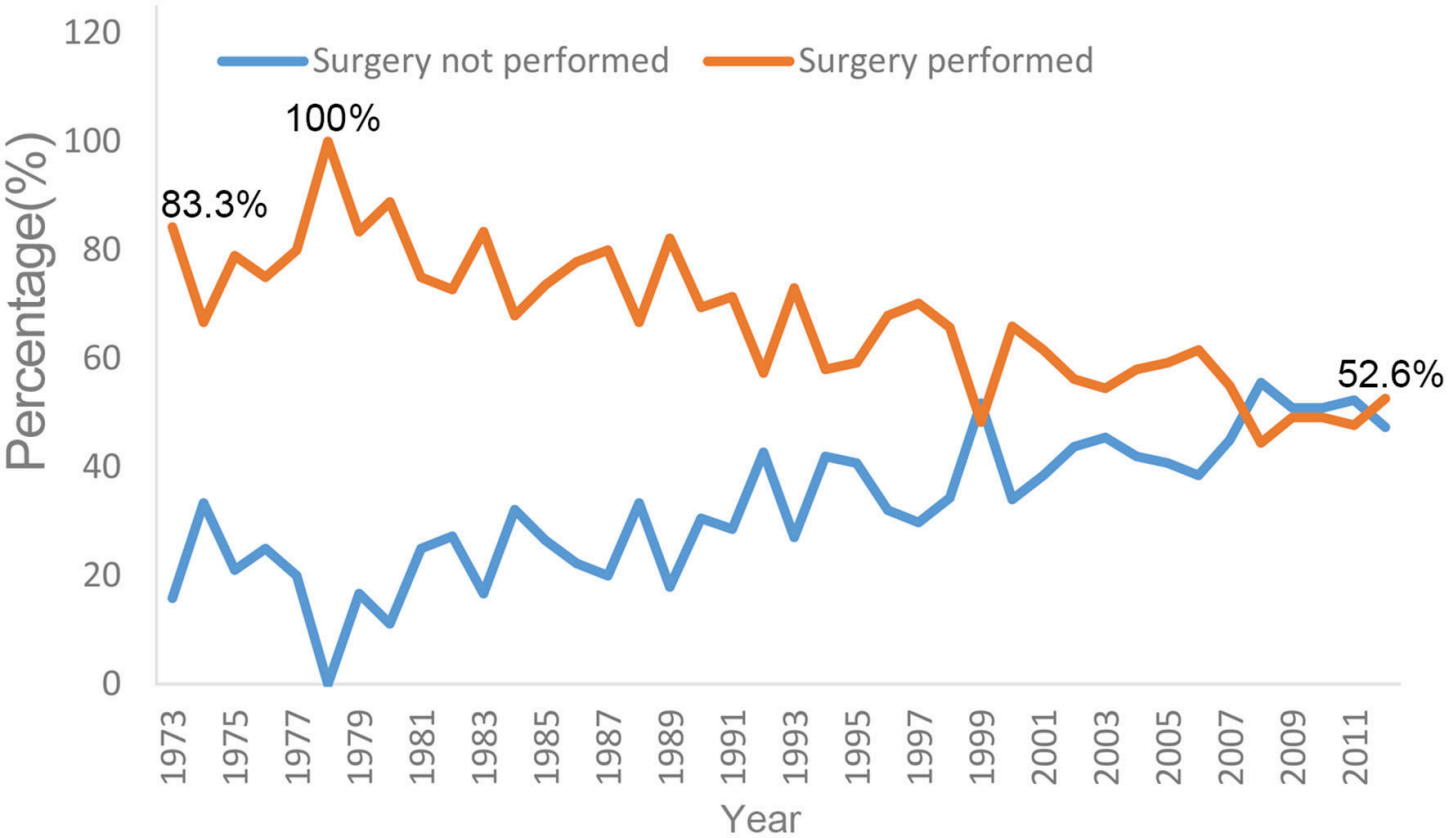
Trends in surgery and non-surgery therapy in SEER 18 registries, 1973–2012.

The proportion of surgical resection changed markedly from 1973 to 2012 (Figure 3). From 1978 to 1980, 83.3–100% of affected patients underwent surgery. After 1980, the proportion of patients receiving surgery gradually decreased. From 2010 to 2012, the proportion of patients who received local resection at diagnosis decreased to 47.7–52.6%. Conversely, the proportion of patients receiving non-surgical therapy increased gradually; this proportion first exceeded the surgical resection rate in 2008 (Figure 3).

### PCL 5-Year Cause-Specific Survival

**Figure 4:**
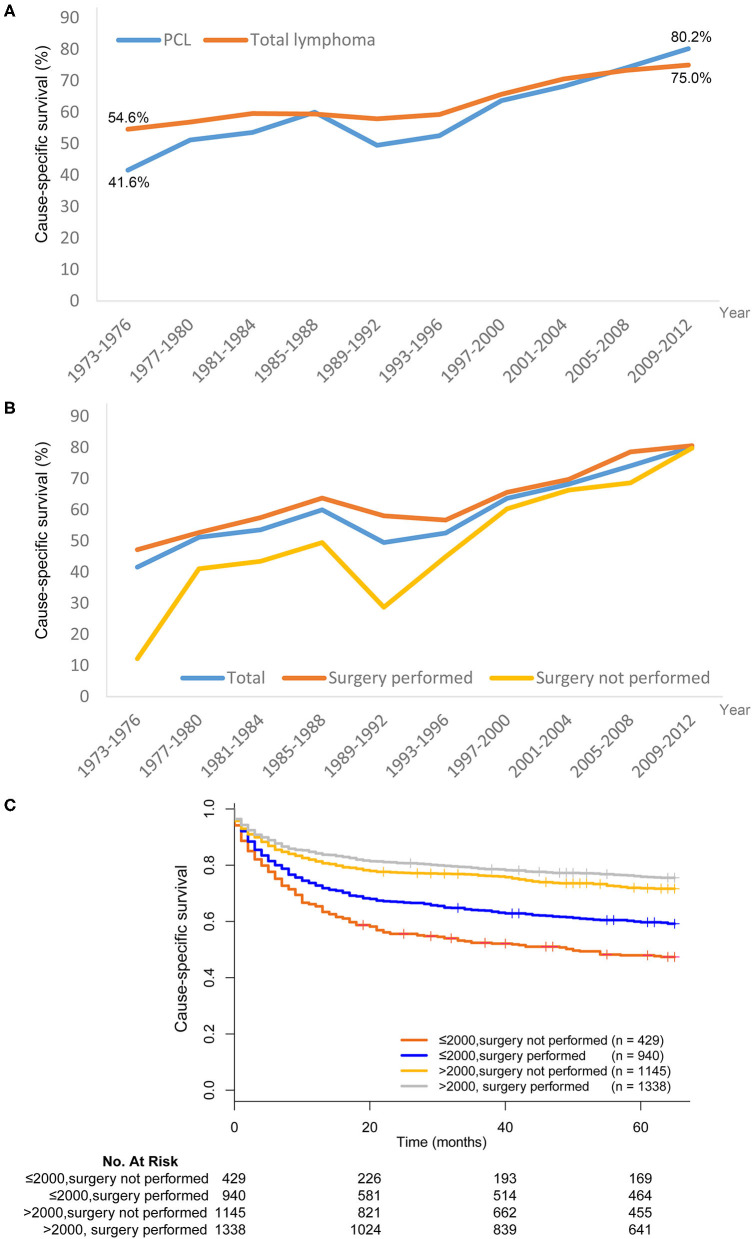
Trends in cause-specific survival (CSS) of lymphoma in SEER 18 registries, 1973–2012. **(A)** The CSS was improved in both primary colorectal lymphoma (PCL) and total lymphoma. **(B)** Patients who received surgical resection of PCL generally had better CSS than those without surgical resection. **(C)** The superiority of surgical resection was significantly reduced after year 1997–2000.

The 5-year cause-specific survival (CSS) for PCL improved markedly from 1973 to 2012. For cases diagnosed between 1973 and 1976, the 5-year CSS was 41.6%, increasing to 80.2% for cases diagnosed between 2009 and 2012. Improved survival was also seen for systemic lymphoma patients, increasing from 54.6 to 75.0% over this time period (Figure 4A). There was no apparent difference in CSS between PCL and systemic lymphoma.

Patients who received surgical resection of PCL generally had better CSS than those who did not receive surgical resection (Figure 4B). However, the superiority of surgical resection was significantly reduced after the 1997–2000 time period. We next divided patients into two subgroups–those who underwent surgical resection before or after 2000. In the period of 1973–2000, there was an absolute 11.7% improvement in the 5-year CSS for patients treated with surgical resection compared with those who did not undergo surgical resection (59.7 vs. 48.0%, χ^2^=15.268, *P* < 0.001). For the period of 2001–2012, although the survival difference was still statistically significant, there was only a 4.1% improvement in 5-year CSS in patients who received surgical resection (75.9 vs. 71.8%, χ^2^ = 5.373, *P* = 0.020) (Figure 4C).

## Discussion

Lymphoma involving the colon and rectum is rare and can occur either as primary colorectal lymphoma or as a manifestation of metastasis (2). Owing to its low incidence rate, PCL has not been well-described. To our knowledge, this is the largest contemporary study describing the incidence, initial local therapy, and outcomes of PCL. In our analyses of data spanning 42 years, the overall incidence of PCL increased from 1.4 to 3.5 per 1,000,000 from 1973 to 2014. The incidence of PCL in older people was significantly higher than in younger people. The incidence increased from 5.1 to 14.1 per 1,000,000 elderly people, while it increased from 0.6 to 1.4 per 1,000,000 young people in the studied time period. The incidence of systemic lymphoma also gradually increased, showing a trend similar to that of PCL.

In the earlier years included in this study, surgical resection was generally recommended to treat PCL in the absence of metastasis (10, 15–18). However, based on pathological examinations of these patients, only 14–24% had no regional lymph node metastases (stage IE), while the majority of patients (ranging from 62 to 86%) had regional lymph node involvement (stage IIE). Moreover, a small number of patients were ultimately found to have diffuse visceral involvement at the time of laparotomy (stage IVE) (2). These findings suggest that PCL is a systemic disease, indicating that surgery may not be the best treatment for PCL. Some authors have stated that it is beneficial to perform colectomy to prevent spontaneous perforation (5), a complication that has a high rate of occurrence. However, others believe that early diagnosis and timely onset of chemotherapy might be adequate treatments for such patients, thus avoiding surgical intervention (19). Indeed, chemotherapy is effective for treating PCL. The CHOP chemotherapy regimen (cyclophosphamide, doxorubicin, vincristine, and prednisone) is the standard first-line therapy for B-cell lymphomas. Rituximab, which was approved by the Food and Drug Administration (FDA) in 1997, has led to high response rates and good progression-free, disease-free and overall survival (20–22). In the present study, we found that the proportion of different tumor stages was not changed over the study period, but the 5-year CSS increased steadily. Conversely, the rate of surgical resection decreased significantly over the study period. These results suggest that the improved survival of PCL patients may be primarily caused by the advancement of chemotherapies and biotargeted therapies. CHOP combination chemotherapy has cured 30% of patients with diffuse large-cell lymphomas (23). Second- and third-generation regimens with predicted 5-year survival rates of >55% have been recently developed (24–26). The addition of rituximab to the CHOP regimen has significantly increased the complete response rate and has prolonged event-free and overall survival (20–22).

This study was limited by the extent of information available in the SEER database. No information on therapeutic strategies was available, so we cannot directly assess the impact of this increasingly important treatment parameter. Furthermore, we do not know the type of surgery performed. As the majority of PCLs have lymph node metastases, it is unknown whether extensive lymphadenectomy offers a survival benefit. The unifying category of lymphoma was first proposed in 1994 (13, 14), which leads to the objective difficulty in recognizing lymphomas prior to 1994, resulting in the possible underestimation of incidence before this time. Finally, the SEER database lacks information on the education, income status, insurance status, and marriage status of patients, which have been validated as prognostic factors (27, 28), and we could not adjust for these factors in the survival analysis.

## Conclusion

PCL remains a rare form of extranodal lymphoma. The data presented here demonstrated the increasing incidence of PCL throughout the study period. Concurrently, the PCL survival outcomes increased continuously over this time period, while the surgical resection rate decreased steadily. Changes in the survival trends and therapy strategies indicate that PCL can be well-managed with newer therapeutic reagents. Additional investigations should be carried out to further verify the interesting findings presented here.

## Data Availability Statement

All datasets generated for this study are included in the article/Supplementary Material.

## Ethics Statement

Ethical approval was not provided for this study on human participants because this study was completely based on the publicly available SEER database and we have got the permission to access them on purpose of research only. It did not include interaction with humans or use personal identifying information. The informed consent was not required for this research. Written informed consent for participation was not required for this study in accordance with the national legislation and the institutional requirements.

## Author Contributions

QL, SM, and WD planned the study. SM and WD calculated statistics and analyzed the data. YL and QL wrote the manuscript. XL, SC, and GC supervised the entire project. All authors reviewed the manuscript.

## Conflict of Interest

The authors declare that the research was conducted in the absence of any commercial or financial relationships that could be construed as a potential conflict of interest.

## Abbreviations

CRC: colorectal cancer
SEER: the Surveillance, Epidemiology, and End Results
PCL: primary colorectal lymphoma
NHL: non-Hodgkin's lymphoma
DLBCL: Diffuse large B-cell lymphoma
MZL: marginal zone lymphoma
MALT: mucosa-associated lymphoid tissue
CHOP, cyclophosphamide, doxorubicin: vincristine and prednisone
FDA: Food and Drug Administration
CSS: cause-specific survival.
